# Novel Zn^2+^ Modulated GPR39 Receptor Agonists Do Not Drive Acute Insulin Secretion in Rodents

**DOI:** 10.1371/journal.pone.0145849

**Published:** 2015-12-31

**Authors:** Ola Fjellström, Niklas Larsson, Shin-ichiro Yasuda, Takuma Tsuchida, Takahiro Oguma, Anna Marley, Charlotte Wennberg-Huldt, Daniel Hovdal, Hajime Fukuda, Yukimi Yoneyama, Kazuyo Sasaki, Anders Johansson, Sara Lundqvist, Johan Brengdahl, Richard J. Isaacs, Daniel Brown, Stefan Geschwindner, Lambertus Benthem, Claire Priest, Andrew Turnbull

**Affiliations:** 1 Medicinal Chemistry CVMD iMed, AstraZeneca R&D Gothenburg, Mölndal, Sweden; 2 Discovery Sciences, AstraZeneca R&D Gothenburg, Mölndal, Sweden; 3 Pharmacology Research Laboratories II, Mitsubishi Tanabe Pharma Corporation, Kawagishi, Toda-shi, Saitama, Japan; 4 Discovery Sciences, AstraZeneca R&D, Mereside, United Kingdom; 5 Bioscience CVMD iMed, AstraZeneca R&D Gothenburg, Mölndal, Sweden; 6 DMPK CVMD iMed, AstraZeneca R&D Gothenburg, Mölndal, Sweden; 7 DMPK Research Laboratories, Mitsubishi Tanabe Pharma Corporation, Kawagishi, Toda-shi, Saitama, Japan; 8 Molecular Sensing, Inc., Nashville, Tennessee, United States of America; 9 CVMD iMed, AstraZeneca R&D Gothenburg, Mölndal, Sweden; Institut d'Investigacions Biomèdiques August Pi i Sunyer, SPAIN

## Abstract

Type 2 diabetes (T2D) occurs when there is insufficient insulin release to control blood glucose, due to insulin resistance and impaired β-cell function. The GPR39 receptor is expressed in metabolic tissues including pancreatic β-cells and has been proposed as a T2D target. Specifically, GPR39 agonists might improve β-cell function leading to more adequate and sustained insulin release and glucose control. The present study aimed to test the hypothesis that GPR39 agonism would improve glucose stimulated insulin secretion *in vivo*. A high throughput screen, followed by a medicinal chemistry program, identified three novel potent Zn^2+^ modulated GPR39 agonists. These agonists were evaluated in acute rodent glucose tolerance tests. The results showed a lack of glucose lowering and insulinotropic effects not only in lean mice, but also in diet-induced obese (DIO) mice and Zucker fatty rats. It is concluded that Zn^2+^ modulated GPR39 agonists do not acutely stimulate insulin release in rodents.

## Introduction

T2D occurs when the pancreatic β-cell can no longer release sufficient insulin to control blood glucose, due to insulin resistance in liver, muscle and adipose tissue. The mechanisms leading to β-cell failure remain debated. However, high levels of glucose and fatty-acids (glucolipotoxicity) activate pathways related to endoplasmic reticulum stress, oxidative stress, mitochondrial dysfunction, apoptosis and islet inflammation which contribute to loss of functional β-cell mass [1]. Agents that will restore insulin secretion by counteracting these effects are therefore of interest as potential therapies for T2D. Glucose is the main physiological trigger for insulin release and this insulinotropic effect is enhanced by other factors that signal through G-protein-coupled receptors (GPCRs), a number of which are expressed in the pancreatic islet [2]. Some islet GPCRs activate the Gα_q_ pathway with subsequent activation of phospholipase C and others activate Gα_s_, which increases intracellular cAMP, both leading to increased insulin secretion. GPCRs therefore represent promising targets for therapy in T2D, with potential to improve β-cell function.

GPR39 is a GPCR that is mainly expressed in peripheral tissues with important metabolic activity, such as pancreatic β-cells, liver, gastrointestinal tract, and adipose tissue [3–5]. Zn^2+^ has been shown to be a stimulator of GPR39 activity via the Gα_q_, Gα_s_ and Gα_12/13_ pathways and has been suggested to be an important physiological regulator of the receptor [6,7]. A number of studies into effects of GPR39 modulation suggest a role of the receptor in glucose homeost{{}}asis and particularly β-cell function. A number of these suggest an overall protective role of GPR39 activity. For example, GPR39 knock-out mice have a reduced glucose tolerance and reduced insulin release in response to glucose challenge in mice fed a variety of different diets [4,8]. Consistent with this protective effect, GPR39 overexpression in β-cells, protects against the gradual hyperglycemia observed after low-dose STZ treatment of mice [9]. However, other studies have suggested a protective role of loss of GPR39 function in rodents [10–12].

To clarify the role of endogenous GPR39 and identify the potential for pharmacological modification of GPR39 activity to modify glucose-stimulated insulin secretion and be a potential treatment for T2D, we have identified potent and selective GPR39 agonists and tested their acute insulinotropic effects in normal mice and in rodent models of T2D.

## Materials and Methods

### Preparation of AZ7914, AZ4237, AZ1395

6-(3-chloro-2-fluoro-benzoyl)-2-(2-methylthiazol-4-yl)-3,5,7,8-tetrahydropyrido-[4,3-d]pyrimidin-4-one (AZ7914), 6-[(4-chlorophenyl)methyl]-7-hydroxy-5-methyl-pyrazolo[1,5-a]pyrimidine-3-carboxylic acid (AZ4237), 3,4-bis-(2-imidazol-1-ylethoxy)-benzonitrile (AZ1395), were prepared as described in the Supporting Information section (S1 File).

### Cloning of GPR39 and cell culture

Full length human (AF034633), rat (DM091294) and mouse (BC085285) GPR39 (GeneArt–Life Technologies, Carlsbad, CA) were subcloned into the pIRESneo3 vector (Clontech, Mountain View, CA). The resulting expression vectors hGPR39-, rGPR39- and mGPR39-pIRESneo3 were transfected into HEK293s cells (ATCC CRL-1573) using Lipofectamine 2000 (Life Technologies). Individual clones were isolated using flow cytometry with a FACSVantage SE DIVA (Becton Dickinson, Franklin Lakes, NJ) and clones were chosen based on responses to Zn^2+^ measured in a cAMP assay (see below). Similar to results by Holst *et al*. [7,13], clones expressing GPR39 displayed constitutive activity in inositol phosphate assays (up to 30% of maximal Zn^2+^ stimulated activity) but not in cAMP assays. HEK293s-derived cell lines were cultured at 37°C, 10% CO_2_ and 95% humidity in DMEM supplemented with 10% FBS and 600 μg/ml Geneticin (Life Technologies). The mouse pancreas epithelial cell line NIT-1 (ATCC CRL-2055) with endogenous expression of GPR39 was cultured at 37°C, 5% CO_2_, and 95% humidity in Ham´s F12 (Life Technologies) supplemented with 10% FBS.

### cAMP assay

Measurements of cAMP production were performed using homogeneous time resolved fluorescence (HTRF) cAMP dynamic kits (CisBio, Codolet, France). All buffers and reagents were according to manufacturer’s protocol. In short, 0.1 μl of compounds in DMSO were added to small-volume 384-well plates (Greiner, Frickenhausen, Germany). 12,000 (5 μl) cells in Hank’s balanced salt solution (HBSS) (Life Technologies) and 20 mM HEPES (pH7.4; Life Technologies) were added per well. The production of cAMP was stimulated for 75 min at 37°C by addition of 5 μl 3-isobutyl-1-methylxanthine (0.375 mM final concentration; Sigma-Aldrich, St Louis, MO) and ZnCl_2_ (0 to 30 μM; Sigma). The reaction was stopped by addition of 5 μl cAMP-d2 and 5 μl anti-cAMP cryptate. The cAMP production was detected by HTRF (λ_ex_ = 340 nm, λ_em_ = 665 and 615 nm) using a Pherastar (BMG Labtech, Ortenberg, Germany).

### IP_1_ assay

IP_1_ assays were performed using IP-One Tb kits (CisBio). IP_1_ production was detected by HTRF as described above. Buffers and reagents were according to manufacturers’ protocols except LiCl concentrations in stimulation buffer which were 50 mM in assays employing HEK293s-hGPR39 cells and 20 mM in assays using NIT-1 cells. For assays using HEK293s-hGPR39 cells, 12,500 cells per well were dispensed into small-volume 384-well plates (Greiner) and IP_1_ production was stimulated in the presence of compounds for 30 min at 37 ˚C in a total volume of 20 μl. For assays using NIT-1 cells, 25,000 cells per well were seeded into poly-D-lysine coated 384-well plates (Becton-Dickinson) and cultured in Ham´s F12 medium with 10% FBS. After 48 h, cells were washed with stimulation buffer and IP_1_ production stimulated in the presence of compounds for 45 min at 37 ˚C in a total volume of 60 μl.

### Dynamic mass redistribution (DMR)–label-free assay

DMR assays were performed using an Epic instrument (Corning Inc., Corning, NY). 12,000 cells per well were cultured for 24 h in 384-well fibronectin-coated Epic biosensor plates (Corning). On the day of experiment, culture medium was removed and replaced with 30 μl buffer A (HBSS, HEPES 20 mM pH7.4, 1% DMSO, 0.01% BSA) using an EL406 (Bio-Tek). Plates were incubated at 26 ˚C for 1 h inside the Epic to allow for temperature equilibration. A baseline was read for 2 min, and then 15 μl compounds in buffer A were added using a CyBi-Well vario (CyBio) and DMR was detected during 45 min.

### Back-scattering interferometry (BSI) binding assay

Membranes (1.3 mg protein/ml) from HEK293s-hGPR39 and untransfected HEK293s cells were diluted 11-fold in 50 mM Tris, pH 7.5, 5 μM ZnCl2, 0.3% DMSO. The compounds were diluted from 10 mM in DMSO to the final maximum stock concentration of 30 μM with 50 mM Tris, pH 7.5, 5 μM ZnCl2, 24.6 mM sucrose. A 3-fold serial dilution was performed with the compounds and refractive index-matched assay buffer, 50 mM Tris, pH 7.5, 5 μM ZnCl2, 24.6 mM sucrose, 0.3% DMSO. The compound serial dilutions were mixed 1:1 with either the GPR39 or wild type membrane fractions in a 96-well polypropylene microplate (Eppendorf, Hauppauge, NY). After a 2 hour incubation at room temperature, the plate was analyzed on a pre-commercial prototype BSI instrument (Molecular Sensing, Inc., Nashville, TN) [14]. Binding signal was expressed as the phase shift in the interference fringe patterns on a CMOS camera as measured in milliradians. The data from the wild type membrane fractions was subtracted from the GPR39-expressing fractions point by point to obtain the specific binding interaction of the compounds with GPR39 and fit to one-site binding or two-site binding models using built-in models in Graphpad Prism to determine *K*_D_ values for each compound.

### Isothermal calorimetry (ITC)

To determine the binding affinities of the discussed compounds for Zn^2+^, isothermal titration calorimetry experiments were conducted on a MicroCal ITC-200 system (Malvern Instruments) at a temperature of 30°C. The compounds, provided as a DMSO stock solution of 10 mM, were diluted in 50 mM HEPES (pH 7.4) and 150 mM NaCl to reach a final nominal concentration of 400 μM (= 4% (v/v) final DMSO concentration). Complete titration of the compounds was typically achieved by injecting 27 x 1.3 μl aliquots of 5 mM ZnCl_2_ provided in a matching buffer. The thermodynamic binding parameters were extracted by non-linear regression analysis of the binding isotherms (Microcal Origin version 7.0 software package). A single site binding model was applied, yielding binding enthalpy (ΔH), stoichiometry (n), entropy (ΔS), and association constant (K_a_). All experiments have been performed in triplicate to allow for error estimations.

### Calculations and statistical analysis

Concentration response data were fitted with a four parameter logistic fit using Eq 1,

$$y=A+((B-A)/1+({\left(C/x\right)}^{D})))$$ (1)

where A is no activation, B is full activation, C is the EC_50_ and D is the Hill slope.

To facilitate visualization of data, efficacy was normalized to % effect of the maximum response stimulated by in each assay.

Concentration response curves for the interaction between ortosteric and allosteric ligands in the DNR assay were globally fitted to the operational model of allosterism and agonism as described in Leach et al. [15]. Binding data were fitted using one site or two site binding models.

### Insulin secretion assays

INS-1E cells obtained from Dr. Wollheim (Univ. Geneva, Switzerland), were grown in Roswell Park Memorial Institute medium (RPMI) 1640 (Life technologies) supplemented with 5% of fetal bovine serum (Thermo Fischer Scientific, Inc. Waltham, MA). At 4 or 5 days before the assay, cells were plated in 24-well plates at 2 × 10^5^ cells /well. At the day of the assay, cells were washed twice by Krebs-Ringer bicarbonate (KRB) buffer containing 0.1% BSA (pH7.4) and pre-incubated at 37°C for 30 min. The cells were washed twice again, and the buffer was replaced by KRB buffer containing 0, 1, 3 and 10 μM of compounds and 11.1 mM of glucose. In order to examine whether Zn^2+^ has an enhancing effect on the activity of each compound, 20 μM of Zn^2+^ was added in the incubation buffer as necessary. After cells were stimulated at 37°C for 1 h, the reaction was terminated by placing on ice. The buffer was collected from each well and precipitated (1000 rpm for 2 min at 25°C) in order to be separated from the cells. For each sample, concentration of insulin was measured using HTRF insulin kit (Cisbio).

### Insulin secretion in mouse islets

C57BL6 mice were humanely killed according to UK Home Office–approved procedures and islets were isolated as described in Johnson *et al*. [16]. Islets were then handpicked and cultured overnight in RPMI 1640 (5 mM glucose; Invitrogen, Paisley, Scotland, U.K.), 10% FCS (Invitrogen), and penicillin/streptomycin (100 units/ml, 0.1 mg/ml; Invitrogen) at 37°C in a 5% CO_2_ humidified atmosphere. The islets were handpicked in groups of three and incubated in HEPES-balanced Krebs-Ringer phosphate (KRH) buffer, composed of 129 mM NaCl, 5 mM NaHCO_3_, 4.8 mM KCl, 1.2 mM KH_2_PO_4_, 1.2 mM MgSO_4_, 10 mM HEPES, 2.5 mM CaCl_2_, 1mM glucose, and 0.1% BSA (pH 7.4, NaOH) for 30min at 37°C and in 5% CO_2_. KRH buffer containing the appropriate glucose concentration and compound was added, and the islets were incubated for 2 h at 37°C and in 5% CO_2_. After incubation, the islets were centrifuged, the medium was collected, and the amount of secreted insulin was determined using HTRF assay (CisBio). Islet data points were obtained from six replicates and repeated three times.

#### Animals

Six-week-old male C57BL/6J mice were purchased from CLEA (Tokyo, Japan). Fourteen-week-old and fifteen-week-old male DIO mice were purchase from Charles River Japan (Yokohama, Japan). They were used after at least one week acclimation period. C57BL/6J mice were allowed free access to a normal diet (CRF-1, Oriental Yeast, Tokyo, Japan). DIO mice were allowed free access to a high-fat diet with 60 kcal% fat (D12492, Research Diets, New Brunswick, NJ). They were kept under standard 12-h light/dark cycle. Animal experiments were approved by the Institutional Animal Care and Use Committee of Mitsubishi Tanabe Pharma Corporation.

### Intraperitoneal glucose tolerance test (IPGTT) in C57BL/6J mice

Seven-week-old and eight-week-old C57BL/6J mice were used. Mice were fasted overnight and were randomly grouped. Five ml/kg of a solution of vehicle (10% dimethylformamide (DMF)/18% Cremophore) or compounds were intraperitoneally (IP) administered. Thirty min later, 1.5 g/20 ml/kg of glucose solution was IP administered. Blood samples were obtained from the tail vein at -30, 0, 15, 30, 60, and 120 min after glucose challenge. Plasma glucose was measured using Glucose CII Test Wako Kit (Wako Pure Chemical Industries, Osaka, Japan). Plasma insulin was measured using Ultra Sensitive Mouse Insulin ELISA Kit (Morinaga, Yokohama, Japan).

### IPGTT in DIO mice

Fifteen-week-old and twenty one-week-old DIO mice were used. The mice were fasted for 3 h and were randomly grouped based on the body weight. Five ml/kg of a solution of vehicle (10% DMF/18% Cremophore) or compounds were administered IP. Thirty min later, 1.0 g/10 ml/kg of glucose solution was administered IP. Blood samples were obtained from the tail vein at -30, 0, 15, 30, 60, and 120 min after glucose challenge. Plasma glucose and insulin were measured.

### Data analysis

Statistical analysis was performed using SAS9.1.3 with student’s t-test or Dunnett’s test. All data were expressed as mean ± standard error of mean (SEM). *P* values of <0.05 were considered to be statistically significant.

### Measurement of unbound compound concentration in plasma

Plasma protein binding of test compound was examined by an equilibrium dialysis method. The compound was mixed with blank mouse plasma to a concentration of 10 μM. The plasma sample and PBS were added to a sample chamber and a buffer chamber of an equilibrium dialysis device, respectively. The device was incubated for 4 h at 37°C in a CO_2_ incubator (oscillation: 180 rpm). After incubation, an aliquot in each chamber was collected. They were mixed with acetonitrile/methanol (50/50, v/v) and were centrifuged. The supernatants were analyzed by LC-MS/MS. Plasma unbound fraction (f) was calculated by Eq 2,

$$\text{f}={\text{C}}_{\text{f}}\;/\;{\text{C}}_{\text{t}}$$ (2)

where C_f_ and C_t_ represent the compound concentration in the buffer and sample chamber, respectively.

### LC-MS/MS analytical procedure for test compounds

Chromatography was performed on an Acquity UPLC system (Waters, Milford, MA) equipped with an Acquity UPLC BEH C_18_ column (30 mm × 2.1 mm, i.d., 1.7 μm particle size, Waters) maintained at 50°C. The mobile phase consisted of 0.025% formic acid (A) and acetonitrile (B). The initial mobile phase was 98% A and 2% B, and the proportion of acetonitrile was linearly increased to 95% over 2.2 min with a flow rate of 0.5 mL/min. An autosampler was set at 8°C with an injection volume of 5 μL. MS analysis was performed on a Waters Xevo TQ-S tandem quadrupole mass spectrometer (Waters) using an electrospray source in positive ion mode. The ionization source parameters were capillary voltage 3.0 kV; source temperature 150°C; desolvation gas temperature 600°C at a flow rate of 1200 L/h; and cone gas flow rate 100 L/h. Nitrogen (99.9% purity) and argon (99.9999% purity) were used as cone and collision gases, respectively. The following selected ion monitoring transitions were used for analysis: 405.04 > 107.90 for AZ7914, 318.06 > 299.81 for AZ4237 and 324.14 > 67.84 for AZ1395, at collision energy of 28, 16 and 34, respectively. Data acquisition was performed by MassLynx Ver. 4.1 software with a QuanLynx program (Waters).

### Calculation of free fraction of drug at in vitro conditions

The free fraction in the INS-1E assay, containing 0.1% w/w BSA, was calculated by using the equilibrium conditions of a single binding site model as in Eq 3.

$$1\;/\;K_{a}=\left[{\text{C}}_{\text{u}}\right]\cdot \left[\text{P}\right]\;/\;\left[\text{CP}\right]$$ (3)

The affinity constant, *K*_*a*_, was first estimated from *f*_*u*_ in mouse plasma (*P*_*tot*_ = 470 μM) by substituting [C_u_] with C_tot_—[CP] and [P] with P_tot_—[CP] and solving the relation for CP. The free fraction in mouse plasma corresponds to 1—CP/P_tot_. The derived *K*_*a*_ is then used to calculate the free fraction in the *in vitro* conditions by using the protein concentration of the assay (~15 μM, Mw~67 kDa).

### Pharmacokinetic-pharmacodynamic (PKPD) analysis

The unbound plasma concentrations were calculated by correcting the total plasma concentration derived from the LC/MS analysis by the free fraction, *f*_*u*_. The exposure in terms of the area under the unbound concentration-time curve in the interval 30–60 min after drug administration *AUCu*_*30–60min*_, *i*.*e*. the interval 0–30 min after glucose challenge, was calculated using the linear up-log down trapezoidal method in the non compartmental module of Phoenix 6.2 (Pharsight). The average concentration in the interval, *C*_*u*,*av*_, was estimated from the ratio of *AUCu*_*30-60min*_ and the time interval. The ratio between *C*_*u*,*av*_ and the *EC*_*50*_ of the different *in vitro* potency assays was calculated for each compound and dose administered.

## Results

### High through-put screen

**Fig 1:**
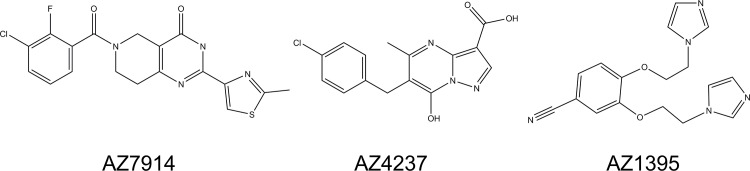
Chemical structures of four GPR39 agonists. AZ7914, 6-(3-chloro-2-fluoro-benzoyl)-2-(2-methylthiazol-4-yl)-3,5,7,8-tetrahydropyrido[4,3-d]pyrimidin-4-one; AZ1395, 3,4-bis(2-imidazol-1-ylethoxy)benzonitrile; AZ4237, 6-[(4-chlorophenyl)methyl]-7-hydroxy-5-methyl-pyrazolo[1,5-a]pyrimidine-3-carboxylic acid.

Approximately 860,000 compounds from the AstraZeneca compound collection were screened in a cAMP assay run in the presence of 5 μM Zn^2+^. After retesting of actives, removal of false positives (actives in GPR39 null cell lines), and confirmation of activity in an orthogonal DMR secondary screen, about 7,000 GPR39 agonists remained. Selection criteria at this stage in the screening cascade considered mainly potency, logD and structural diversity. Representatives from 20 chemical clusters were further characterized with regard to both potency and efficacy measures as those shown in Table 1, as well as absorption, distribution, metabolism and excretion (ADME) parameters such as solubility, intrinsic clearance, CYP inhibition and Caco2 permeability. A limited medicinal chemistry program on selected clusters with a focus on GPR39 potency and ADME parameters in the mouse led to the identification of several clusters from which tool compounds could be derived. Representatives from three different clusters are shown in Fig 1, which were selected for an in depth *in vitro* and *in vivo* characterization.

**Table 1:** *In vitro* characterization of three GPR39 agonists and Zn^2+^ in functional and binding assays.

		AZ7914		AZ4237		AZ1395		Zn^2+^
		Zn^2+^	w/o	Zn^2+^	w/o	Zn^2+^	w/o	-
**DMR**	**hGPR39**	0.10	> 33 (39%)*	0.19	> 33 (20%)*	0.036	2.3	13 (35%)^#^
** **	** **	(6.99 ± 0.29)		(6.72 ± 0.07)		(7.45 ± 0.12)	(5.64 ± 0.12)	(4.87 ± 0.05)
**DMR**	**rGPR39**	0.15	7.4	0.15	> 33 (23%)*	0.033	5.1	
** **	** **	(6.83 ± 0.13)	(5.23 ± 0.03)	(6.83 ± 0.06)		(7.48 ± 0.17)	(5.29 ± 0.09)	-
**DMR**	**mGPR39**	0.32	> 33 (42%)*	0.45	> 33 (12%)*	0.17	10	
** **	** **	(6.50 ± 0.42)		(6.34 ± 0.19)		(6.78 ± 0.30)	(4.98 ± 0.18)	-
**cAMP**	**hGPR39**	2.3	> 33 (21%)*	0.39	> 33 (49%)*	0.49	> 33 (81%)*	12 (60%)^#^
** **	** **	(5.64 ± 0.22)		(6.41 ± 0.20)		(6.31 ± 0.28)		(4.92 ± 0.13)
**IP**_**1**_	**hGPR39**	0.074	> 33 (45%)*	0.034	> 33 (66%)*	0.019	2.9	24 (60%)^#^
** **	** **	(7.13 ± 0.16)		(7.47 ± 0.10)		(7.72 ± 0.17)	(5.54 ± 0.12)	(4.63 ± 0.08)
**IP**_**1**_	**NIT-1**	0.65 (47%)^#^	> 33 (9%)*	0.24 (50%)^#^	> 33 (8%)*	>33 (100%)*	> 33 (0%)*	>33 (0%)*
** **	** **	(6.19 ± 0.17)		(6.62 ± 0.18)				
**BSI**	**hGPR39**	0.192 ± 0.085 (6.72)	-	0.048 ± 0.018 (7.32)	-	0.233 ± 0.070 (6.63)	-	*K*_D1_: 0.53 ± 0.12 (6.27) *K*_D2_: 211 ± 63 (3.68)

**Fig 2:**
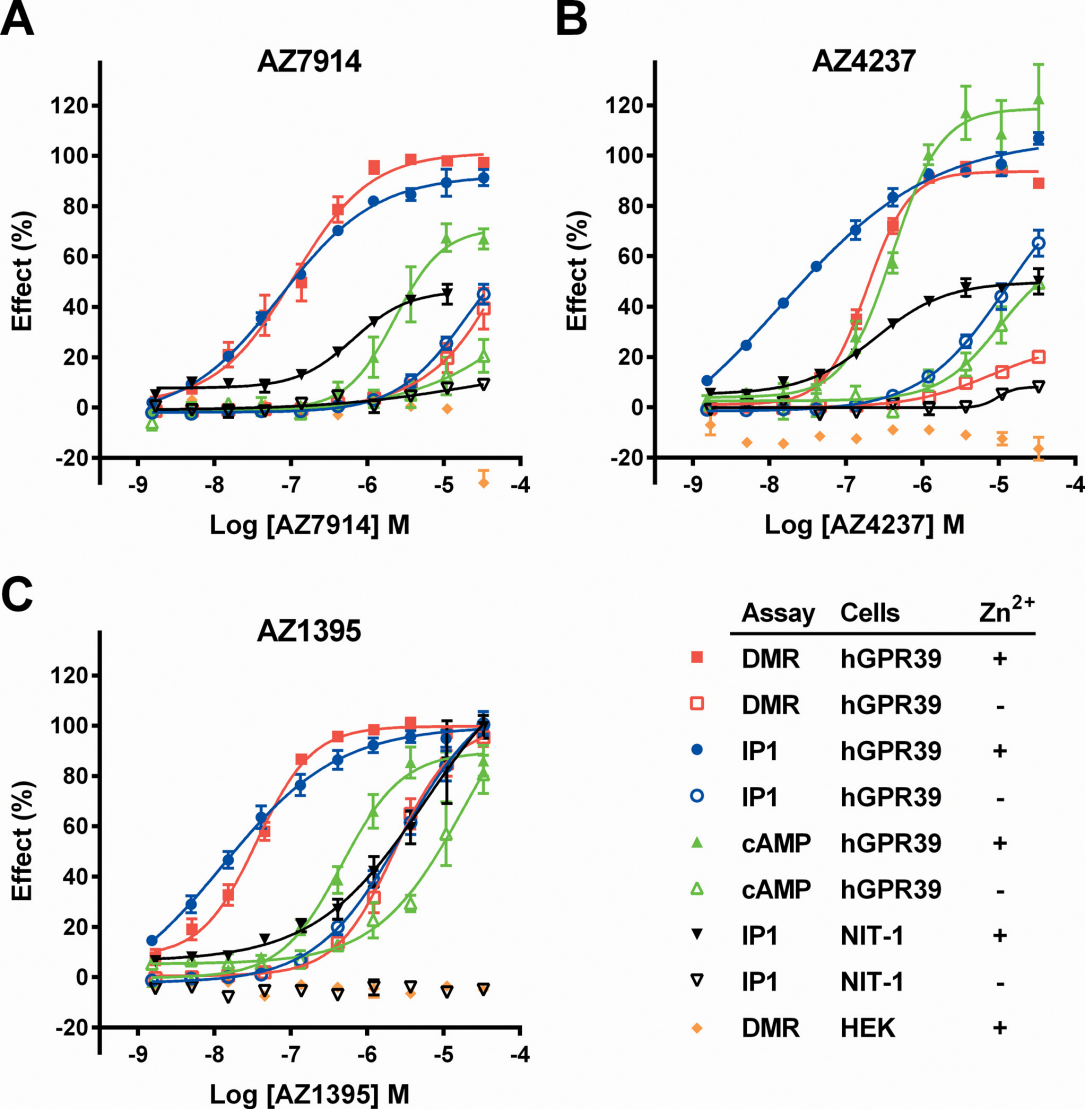
AZ7914 (*A*), AZ4237 (*B*) and AZ1395 (*C*) ten point concentration response curves for *in vitro* screens. Concentration responses of compounds were measured with DMR, IP_1_ and cAMP assays in the absence (-) or presence of 5 μM Zn^2+^ (+) as indicated in the figure. Lines represent fits to Eq 1. Cells employed were HEK293s-hGPR39 (hGPR39), untransfected HEK293s (HEK), and NIT-1 cells endogenously expressing GPR39. Responses were normalized to AZ1395 in each assay and presented as % effect of control. For HEK293s and HEK293s-hGPR39 cells, individual DMR assays were run in singlicates and IP_1_ and cAMP assays were run in triplicates. Values shown are means ± SEM of typically three (two to five) independent experiments. For NIT-1 cells, IP_1_ assays were typically run in duplicates (one to four experiments), and values are shown as means with error bars representing range. EC_50_ values calculated from the data in Fig 2 are summarized in Table 1.

**Fig 3:**
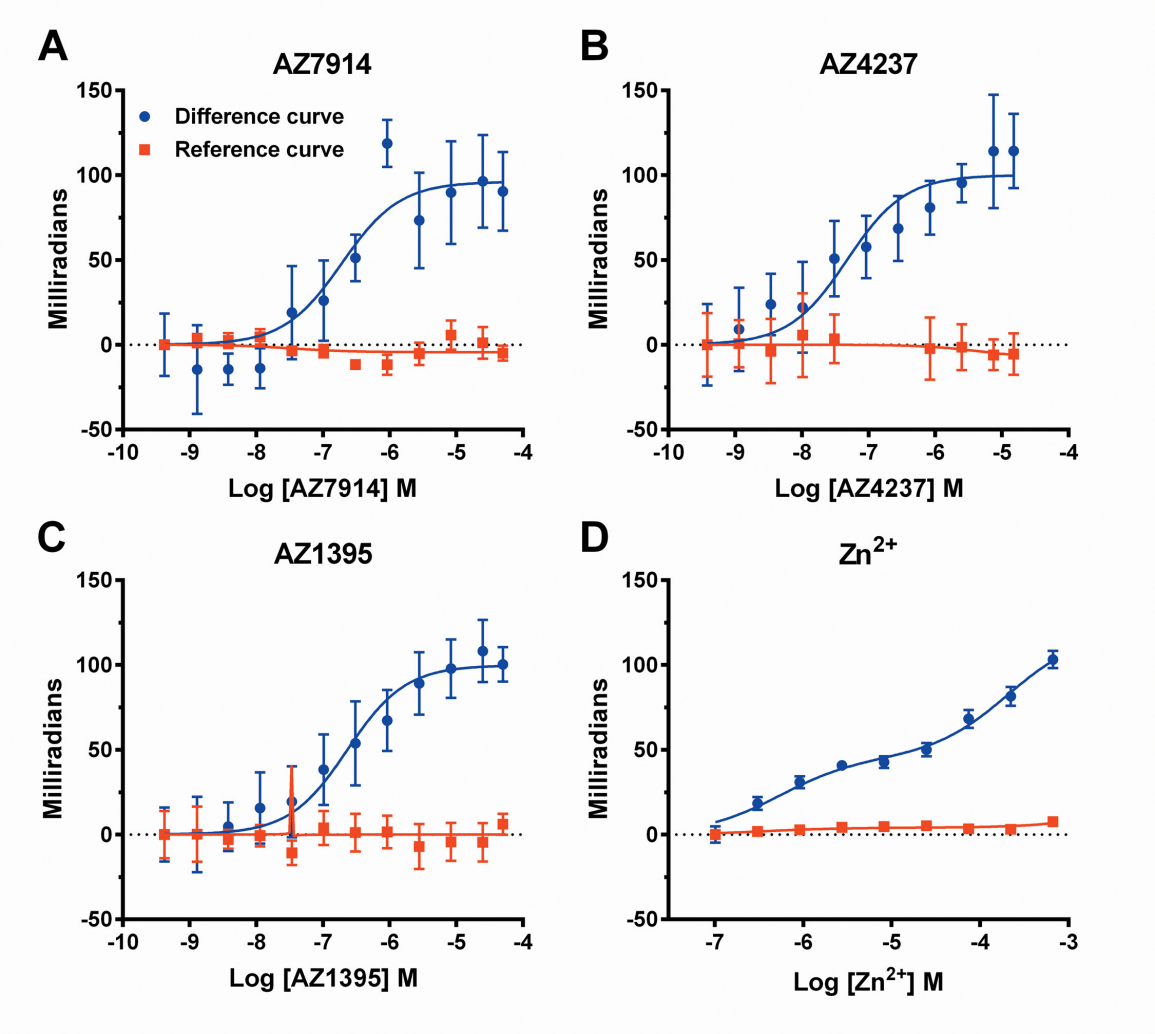
AZ7914 (*A*), AZ4237 (*B*), AZ1395 (*C*) and Zn^2+^ (*D*) concentration response curves in back-scattering Interferometry binding assays. Compounds were incubated with membranes from HEK293s-hGPR39 and HEK293s cells. AZ7914, AZ4237 and AZ1395 were incubated in the presence of 5 μM ZnCl_2_. Signals from HEK293s membranes (reference curves) were subtracted from HEK293s-hGPR39 membrane signals at each concentration point to derive GPR39 specific binding (difference curves). Values shown are means ± SD of three (AZ7914, AZ4237 and AZ1395) or four (Zn^2+^) independent experiments. One-site binding models were used to calculate *K*_D_ values for AZ7914, AZ4237 and AZ1395 summarized in Table 1. For Zn^2+^, data was fitted to a two-site binding model.

EC_50_ values (μM) and pEC_50_ ± SD (shown within brackets) in the absence (w/o) or presence of 5 μM Zn^2+^ in HEK293s cells transfected with human (h), rat (r) and mouse (m) GPR39, and in NIT-1 cells endogenously expressing GPR39. Data is typically from three (two to five) independent experiments. For HEK293s-hGPR39 and NIT-1, calculations were performed on the data shown in Fig 2. Compounds with sufficient potency to reach a plateau in assays employing transfected cells displayed similar efficacy as the AZ1395 100% reference. For compounds not reaching a plateau at highest tested concentration, potencies are shown as >33 μM. For binding affinity measured by BSI, *K*_D_ values (μM) ± SE and p*K*_D_ (within brackets) are shown. Data are from three or four independent experiments and calculation performed on the data shown in Fig 3. AZ7914, AZ4237 and AZ1395 were fitted to a one site binding model and Zn^2+^ to a two site binding model. *K*_D1_ and *K*_D2_ correspond to high and low affinity sites, respectively.

*Efficacy at 33 μM.

^#^E_max_ relative to the 100% efficacy reference AZ1395.

### *In vitro* characterization

GPR39 couples to the Gα_q_, Gα_s_ and Gα_12/13_ pathways [3], and Zn^2+^ is its cognate ligand [6,7]. Therefore, several assays run both in the absence and presence of Zn^2+^ (in general 5 μM) were required for an appropriate profiling of the lead compounds. A summary of the results is presented in Table 1, with selected corresponding concentration response curves shown in Fig 2.

The DMR assay is agnostic of signaling pathway and is thus able to reflect one or several pathways acting simultaneously [17]. All three compounds displayed submicromolar EC_50_ values in the presence of Zn^2+^, which were also in general similar in the corresponding rat and mouse assays. The DMR potencies in the absence of Zn^2+^ were considerably lower for all three compounds, and also indicate variable degrees of Zn^2+^ dependencies.

Zn^2+^ by itself displayed agonism in the three assays with EC_50_ values of 12 μM (cAMP), 24 μM (IP_1_) and 14 μM (DMR). Importantly, the maximum effect stimulated by Zn^2+^ alone was lower than compound-stimulated responses, approximately 60% in cAMP and IP_1_ assays and 35% in DMR assays compared to the maximum response stimulated by AZ1395 together with 5 μM Zn^2+^.

The cAMP and IP_1_ assays capture Gα_s_ and Gα_q_ signaling, respectively. For the compounds, EC_50_ values were between 0.39 and 2.3 μM in the cAMP assay in the presence of Zn^2+^, and were up to 23-fold higher than the corresponding DMR values, see Table 1. In contrast, the IP_1_ and DMR EC_50_ values were more closely matched, which may suggest that the DMR signal mainly reflects Gα_q_ signaling.

The IP_1_ assay was also performed on native mouse NIT-1 β-cells. Zn^2+^ by itself exhibited no agonistic effects in the native NIT-1 cells, despite proven GPR39 expression [8]. All three compounds in the presence of Zn^2+^ displayed potencies in the 1 μM range, and their efficacies varied between 47 and 100%, using AZ1395 as reference agonist. In the absence of Zn^2+^, the compounds were essentially inactive (EC_50_ >33 μM), see Table 1. The enhanced compound potencies in the NIT-1 cells in the presence of Zn^2+^ is an indirect indication that the agonistic effects of the compounds are GPR39 mediated and that the modulating effects of Zn^2+^ remain.

To address if the agonists were directly interacting with GPR39 we employed binding assays based on back-scattering interferometry (BSI) (Fig 3) [14]. Compound binding affinities in the presence of 5 μM Zn^2+^ calculated using one-site binding models are presented in Table 1. BSI also allowed detection of Zn^2+^ binding to GPR39. For Zn^2+^, data fitted best to a two-site binding model with a high-affinity *K*_D_ of 0.53 ± 0.12 μM and low-affinity *K*_D_ of 211 ± 63 μM.

The structures of AZ7914, AZ4237 and AZ1395 suggested that they may potentially directly interact with Zn^2+^. We used isothermal titration calorimetry (ITC) to address this. All three compounds interacted with Zn^2+^ and binding parameters are summarized in Table 2.

**Table 2:** Binding parameters for Zn^2+^-interaction with AZ1395, AZ4237 and AZ7914 determined by ITC at 30°C.

	*K*_D_ (μM)	ΔH (kcal/mol)	n value
**AZ7914**	30.6 ± 19.1	-3.00 ± 0.71	0.56 ± 0.08
**AZ4237**	8.72 ± 1.06	-1.30 ± 0.25	1.18 ± 0.02
**AZ1395**	52.1 ± 9.1	- 4.83 ± 0.29	0.93 ± 0.12

The parameters were extracted from the complete titration of each compound with ZnCl_2_ by the best fit according to a single site binding model, yielding the binding enthalpy (ΔH), stoichiometry (n) and the association constant (*K*_a_), from which the dissociation constant (*K*_D_ = 1/*K*_a_) was calculated. Data are from three experiments ± SD.

**Fig 4:**
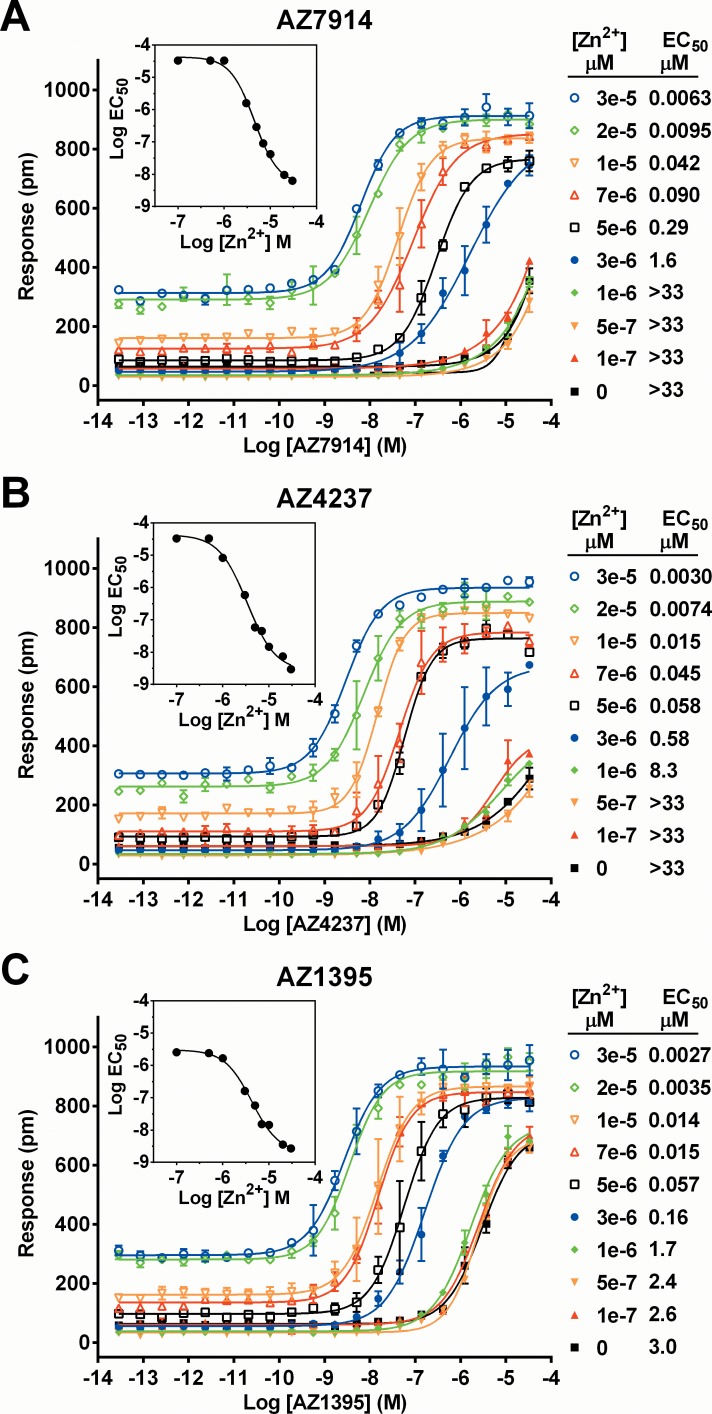
Interdependencies of GPR39 agonists and Zn^2+^; AZ7914 (*A*), AZ4237 (*B*), AZ1395 (*C*), AZ4502 (*D*). Concentration responses of compounds were measured in DMR assays in the absence or presence of fixed concentrations of Zn^2+^ as indicated in the figure. Data are presented as the raw wavelength shifts in pm caused by compound treatment. Lines represent fits to Eq 1. Compound EC_50_ values in the presence of different Zn^2+^ concentration are shown. The experiment was run using duplicate samples and is representative of three independent experiments. Values are means of duplicates with error bars representing range. In the inserts, the obtained compound Log EC_50_ values are plotted as a function of Zn^2+^ concentration and used to calculate the concentration of Zn^2+^ causing half maximum shift of compound EC_50_ curves, *i*.*e*. Zn^2+^ PAM EC_50_ values.

Utilizing the DMR platform, positive allosteric modulator (PAM) experiments were performed in order to more accurately determine the influences of Zn^2+^ on the three compounds, see Fig 4. Compound dose responses were generated at 10 different Zn^2+^ concentrations, spanning 0 to 30 μM. The presence of Zn^2+^ increased potencies of compounds up to 10000-fold. Data were initially globally fitted to the operational model of allosterism and agonism described by Leach et al [15]. However, fits obtained were not of sufficient quality. We therefore decided to calculate modulation in a more simplistic way, here called PAM EC_50_ and defined as the concentration of modulator (Zn^2+^) causing half maximum potentiation of agonist potency. PAM EC_50_ of Zn^2+^ span the range 3.4 to 4.5 μM for the three compounds as illustrated in the inserts in Fig 4.

The same data were analyzed as Zn^2+^ dose response curves at different fixed concentrations of compound. From this reverse analysis, the Zn^2+^ EC_50_ values varied between 19 and 2.1 μM going from 0 to 33 μM fixed compound concentrations. The compound PAM effects, shifting Zn^2+^ EC_50_ up to 7 fold, were moderate compared to the converse Zn^2+^ PAM effects on the AZ compounds. With respect to relative efficacies, E_max_ for Zn^2+^ alone was approximately 35% of E_max_ for Zn^2+^ together with any of the GPR39 agonists. E_max_ for AZ1395 in the absence of Zn^2+^ were approximately 75%. AZ7914 and AZ4237 responses in the absence of Zn^2+^ did not plateau and E_max_ values were therefore not calculated.

**Fig 5:**
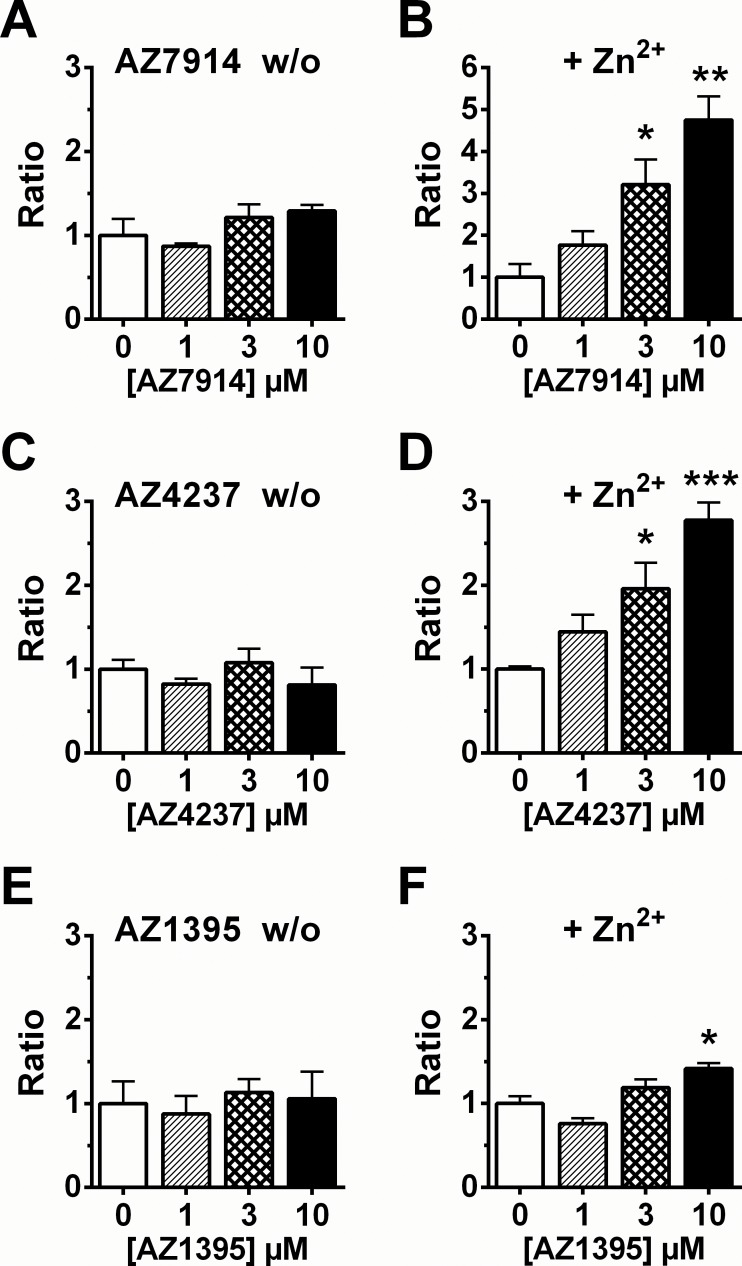
Insulin secretion assays in INS-1E cells. Effect on insulin secretion by each compound. Cells were treated with 1, 3 and 10 μM of AZ7914 (*A*, *B*), AZ4237 (*C*, *D*), AZ1395 (*E*, *F*). The assays were performed in the absence or presence of 20 μM of Zn^2+^. Values were calculated as the ratio of insulin concentration compared to the basal control and expressed as the average of three separate measurements ± SEM. *P<0.05, **P<0.01, ***P<0.001 versus control analyzed by Dunnett.

**Fig 6:**
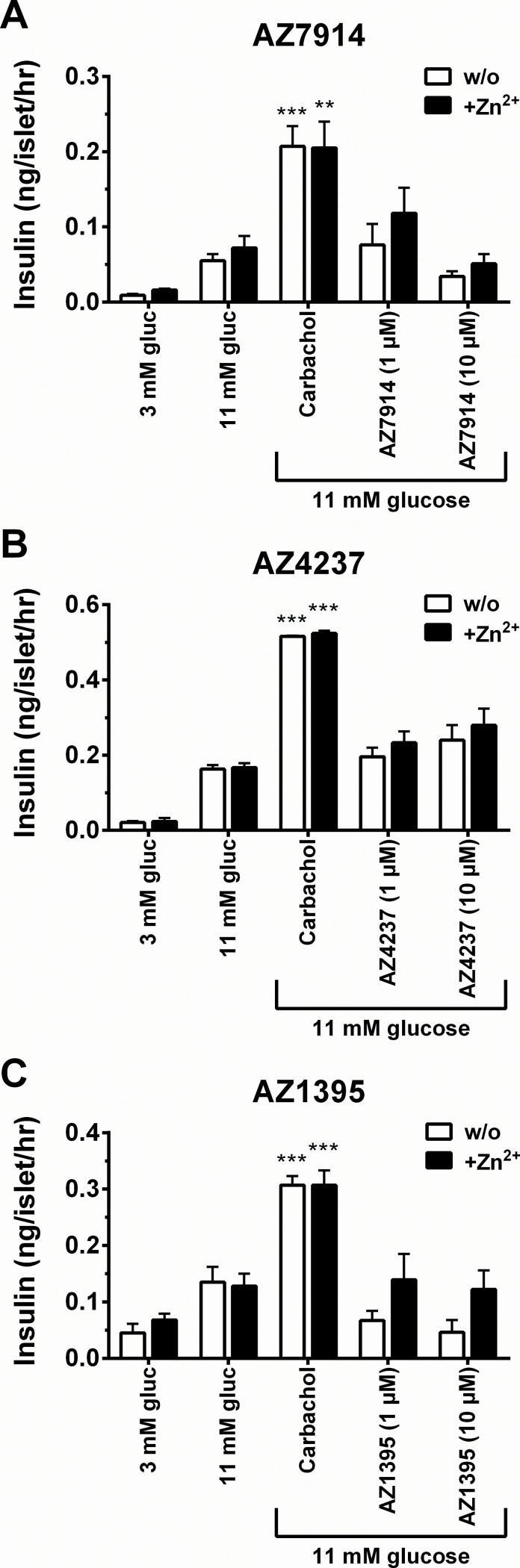
Insulin secretion assays in mouse islets. Effect on insulin secretion by each compound. Islets were treated with 1 and 10 μM of AZ7914 (*A*), AZ4237 (*B*), AZ1395 (*C*). The assays were performed in the absence or presence of 5 μM of Zn^2+^. Values were calculated as the ratio of insulin concentration compared to the basal control and expressed as the average of three separate measurements ± SEM.

Two biological effect assays were developed to measure glucose stimulated insulin secretion (GSIS), one in rat INS-1E β-cells and one in mouse islets. Fig 5 displays the INS-1E data in the absence and presence of Zn^2+^. AZ7914, AZ4237 and AZ1395 all showed increases in GSIS in the presence of Zn^2+^. In the islet GSIS experiments none of the compounds in the presence and absence of Zn^2+^ displayed significantly increased GSIS effects at the 1 and 10 μM compound concentrations tested (Fig 6).

### *In vivo* characterization

**Fig 7:**
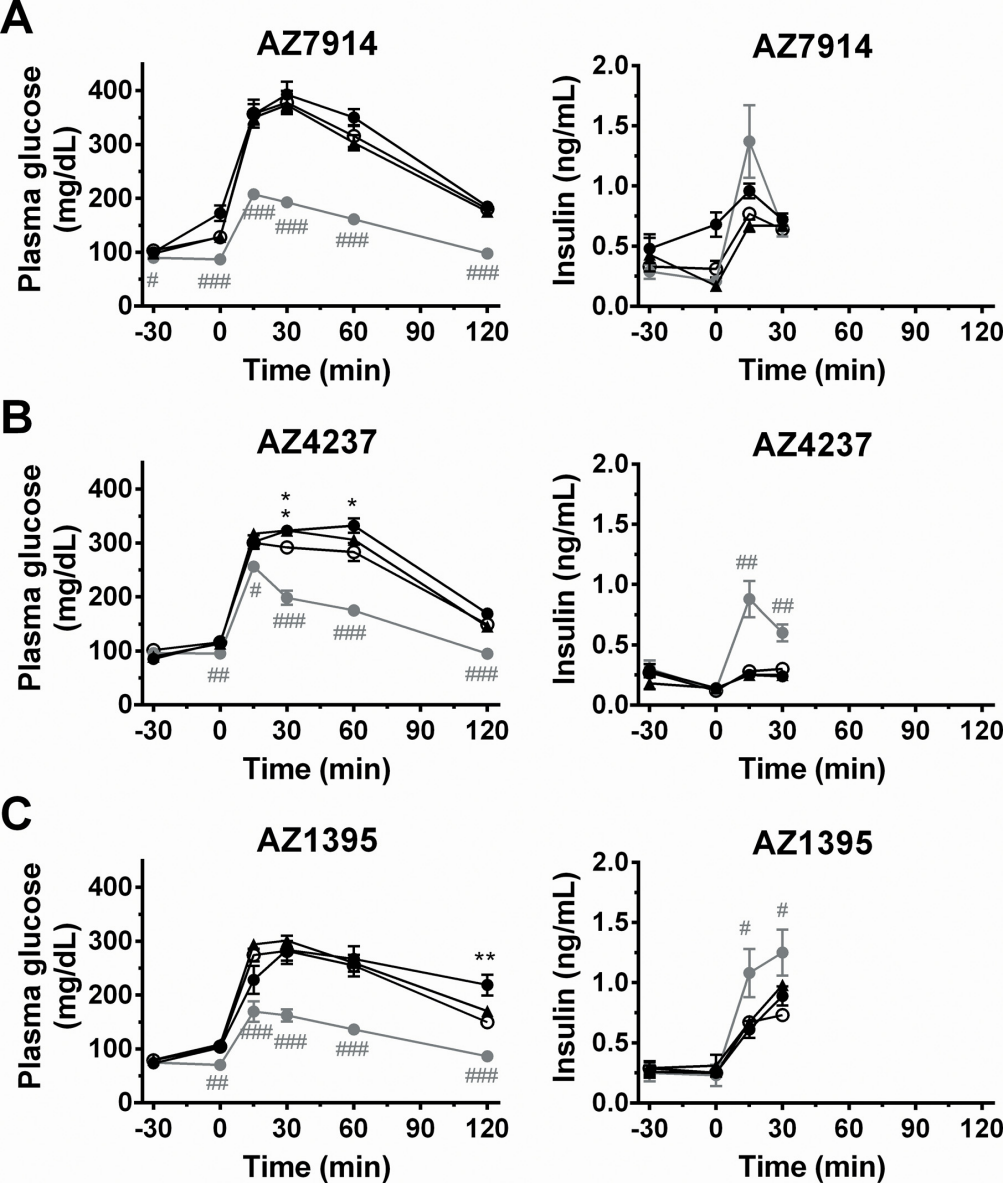
IPGTT in C57BL/6J mice; AZ7914 (*A*), AZ4237 (*B*), AZ1395 (*C*). Compounds (black triangles: 30 mg/kg, black circles: 100 mg/kg, grey circles: exendin-4, 1 μg/kg) or vehicle (white circles) were IP administered to fasted C57BL/6J mice at -30 min. Glucose solution (1.5 g/kg) was given IP at 0 min. Values are mean ± SEM (n = 7). ^#^ *P*<0.05, ^##^ *P*<0.01, ^###^ *P*<0.001 *versus* vehicle (repeated measures ANOVA followed by student’s t-test).*P<0.05, **P<0.01, ***P<0.001 *versus* vehicle (repeated measures ANOVA followed by Dunnett).

IPGTT was chosen as the key experiment for assessing the acute *in vivo* utility of GPR39 agonists. First, the three compounds were tested by IP administration to fasted lean mice 30 min before IP administration of 1.5 g/kg glucose. The GLP-1 agonist exendin-4 [18] was used as positive control, in this and all presented *in vivo* studies. Effect data on glucose and insulin levels are shown in Fig 7. The three agonists showed neither significant glucose lowering effects nor significant insulinotropic effects.

**Fig 8:**
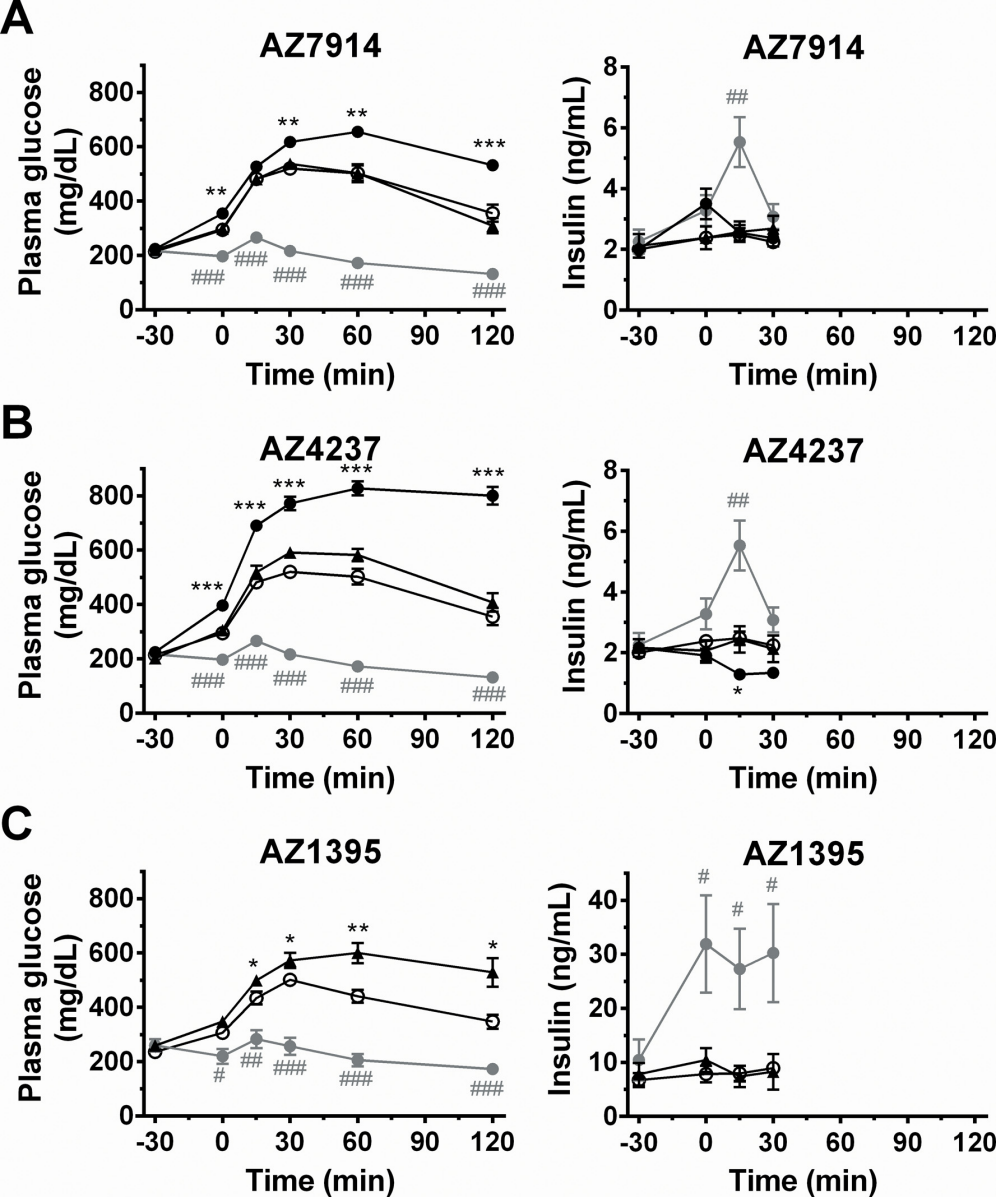
IPGTT in DIO mice; AZ7914 (*A*), AZ4237 (*B*), AZ1395 (*C*). Compounds (black triangles: 30 mg/kg, black circles: 100 mg/kg, grey circles: exendin-4, 1 μg/kg) or vehicle (white circles) were IP administered to fasted DIO mice at -30 min. Glucose solution (1.0 g/kg) was IP given at 0 min. Values are mean ± SEM (n = 7). ^#^ *P*<0.05, ^##^ *P*<0.01, ^###^ *P*<0.001 *versus* vehicle (repeated measures ANOVA followed by student’s t-test).*P<0.05, **P<0.01, ***P<0.001 *versus* vehicle (repeated measures ANOVA followed by Dunnett).

Next, the GPR39 agonists were tested in DIO mouse and in Zucker fatty rat disease models. As shown in Fig 8 for the DIO mouse experiments, none of the agonists showed insulinotropic effects. Moreover, all three compounds yielded significant hyperglycemic effects at their 30 or 100 mg/kg doses. In this study, the 100 mg/kg AZ1395 group was terminated due to severe hyperglycemia and associated morbidity/mortality in some animals. The Zucker fatty rat experiments showed similar trends for the three compounds as shown in S1 Fig.

### PKPD assessment

**Fig 9:**
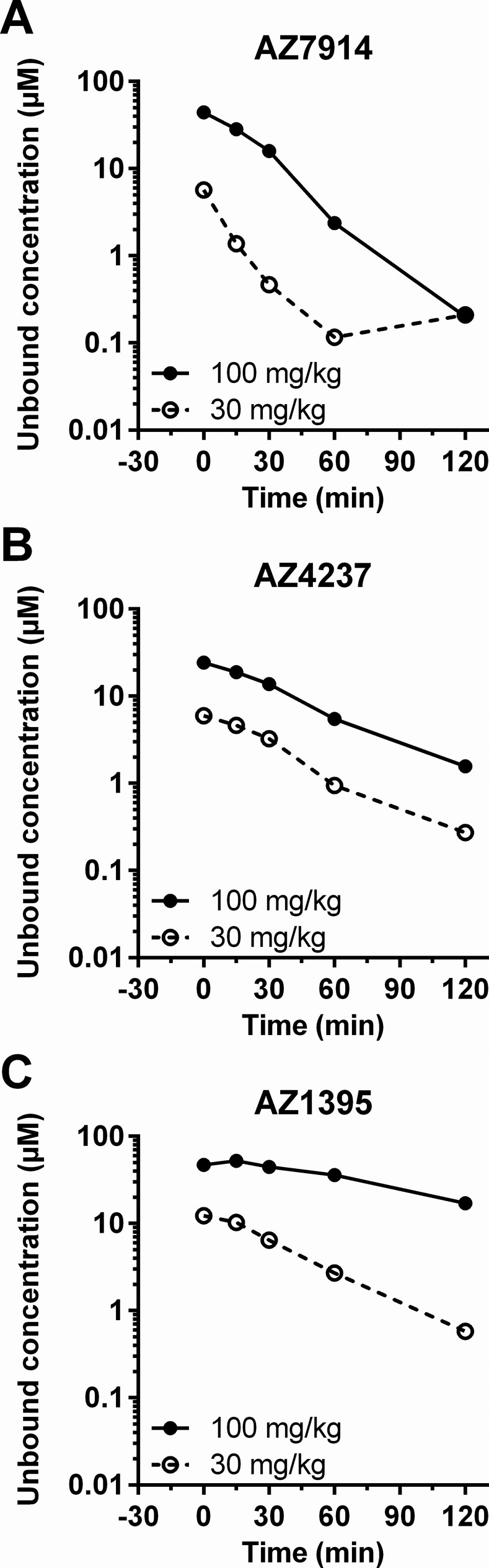
Plasma concentration–time profile of test compounds in the IPGTT using C57BL/6J mice; AZ7914 (*A*), AZ4237 (*B*), AZ1395 (*C*). Compounds were IP administered to fasted C57BL/6J mice at -30 min as described in the Fig 7 legend. Data represent the value of pooled samples from 7 animals.

**Fig 10:**
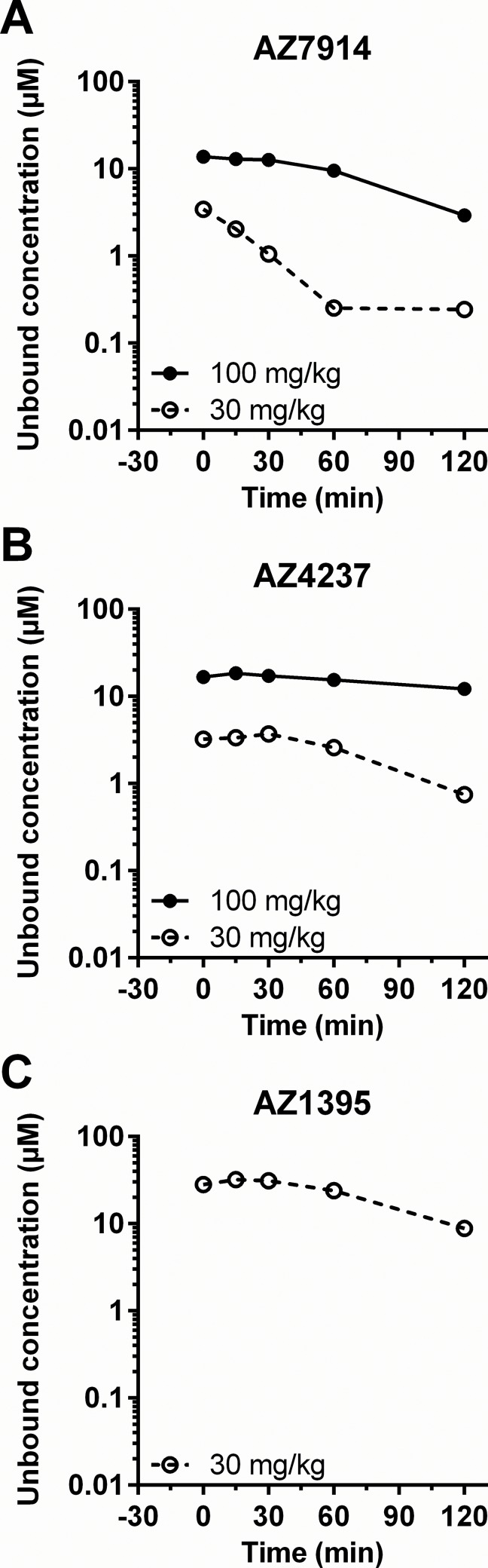
Plasma concentration–time profile of test compounds in the IPGTT using DIO mice; AZ7914 (*A*), AZ4237 (*B*), AZ1395 (*C*). Compounds were IP administered to fasted DIO mice at -30 min as described in the Fig 8 legend. Data represent the value of pooled samples from 7 animals.

**Fig 11:**
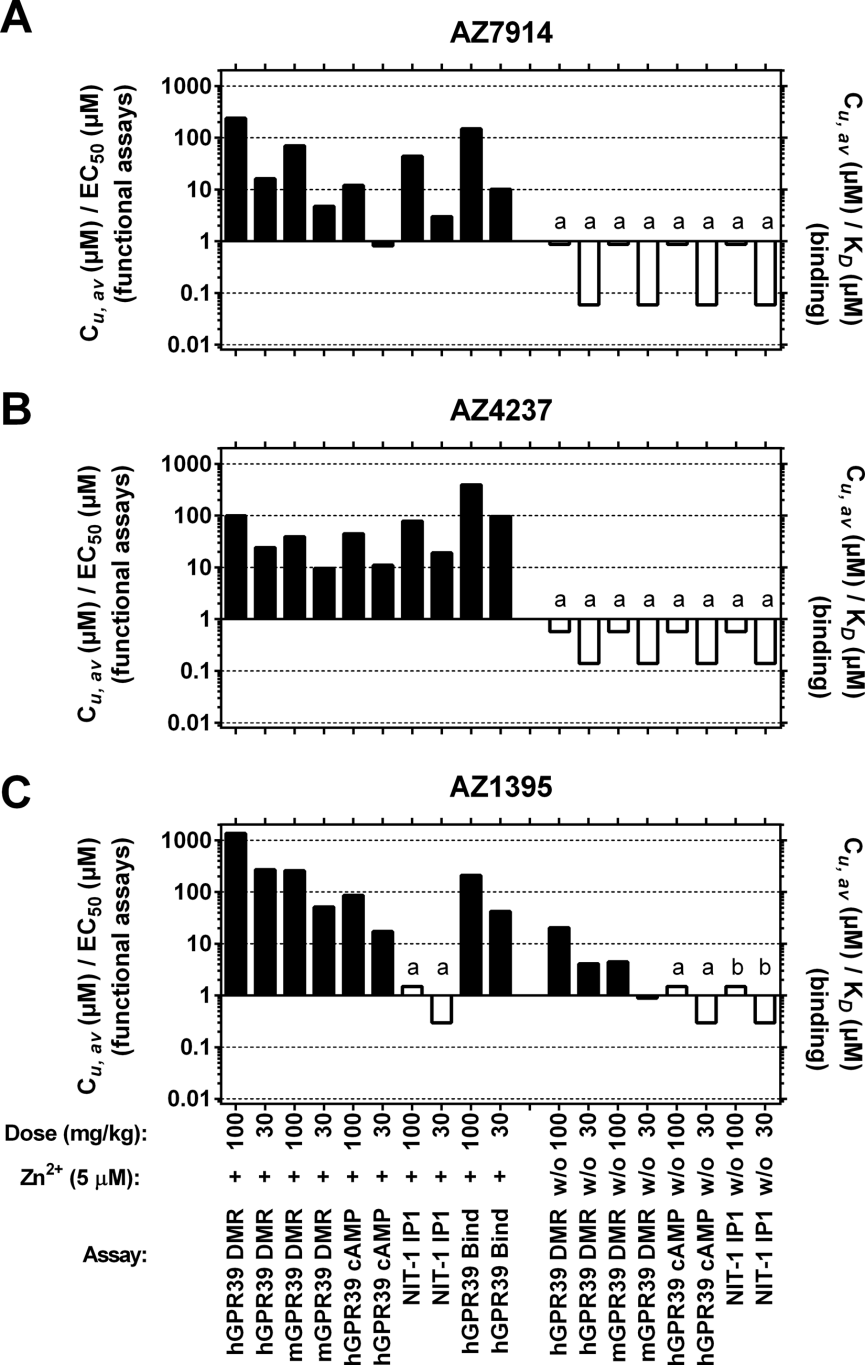
*In vivo* exposures *versus in vitro* potencies for AZ7914 (*A*), AZ4237 (*B*), AZ1395 (*C*). C_*u*_,_*av*_ (μM) / EC_50_ (μM) and C_*u*_,_*av*_ (μM) / *K*_D_ (μM) calculated for different compound doses (30 or 100 mg/kg) IP administered to normal C57BL/6J mice. EC_50_ and *K*_D_ values were from *in vitro* assays (see Table 1) performed in the absence (w/o) or presence of 5 μM Zn^2+^ (+) and adjusted for protein binding. a) Compound weakly active in *in vitro* assay: Potency >33 μM, E_max_ ≥ 8%. b) Compound inactive in *in vitro* assay: Potency >33 μM, E_max_ < 2%. For bars labeled with a) and b), EC_50_ = 33 μM has been used for calculations, resulting in overestimation of C_*u*_,_*av*_ / EC_50_ ratios. This is indicated with open bars. Closed bars indicate that EC_50_ values have been determined to a defined value. ND, not determined.

PK data were generated in order to relate compound unbound exposures in the *in vivo* studies to unbound *in vitro* potency parameters. Plasma concentration-time profiles for each of the three compounds in both C57BL/6J and DIO mice are depicted in Fig 9 and Fig 10, respectively. Dose dependent exposures were seen in both models, although not always a linear dependency, and there was a trend of more durable exposures in the DIO mice compared to the lean mice. The average exposure between 0 and 30 minutes in Fig 9 for each compound in lean mice were calculated and compared to *in vitro* data as shown in Fig 11. AZ7914, AZ4237 and AZ1395 all displayed *in vivo* exposure *versus in vitro* potency ratios >10 for each of the screens in the presence of Zn^2+^, suggesting that none of the DMR, IP1/Gα_q_ or cAMP/Gα_s_ + Zn^2+^ screens were predictive of improved GSIS *in vivo*. In the absence of Zn^2+^, the only *in vivo* exposure *versus in vitro* potency ratio exceeding 10 was for the DMR assay with AZ1395. Thus, from these data on AZ1395 and the lack of positive *in vivo* effects, it seems that DMR *in vitro* potencies in the absence of added Zn^2+^ does not translate to desired *in vivo* effects.

AZ7914 and AZ4237 at unbound concentrations of 8.4 and 7.2 μM, respectively, yielded significant increases in GSIS in INS-1E cells. The average *in vivo* exposures (*C*_*u*,*av*_) of AZ7914 and AZ4237 of 29 and 19 μM, respectively, did not elicit insulinotropic effects *in vivo*, indicating lack of translation of GSIS in INS-1E cells to GSIS *in vivo*.

## Discussion

The aim of the present study was to evaluate effects of GRP39 agonists on insulin secretion and glucose lowering and hence the potential of GPR39 agonism as a therapy for T2D. Three GPR39 agonists derived from HTS were subjected to extensive *in vitro* and *in vivo* profiling and utilized as pharmacological tools for target validation studies. The results show that these Zn^2+^ modulated GPR39 agonists did not improve GSIS neither in normal rodents nor in models of β-cell dysfunction.

The HTS based project work presented here suggests that GPR39 is, in the presence of physiologically relevant levels of Zn^2+^ [19,20], a druggable target, amenable to identification of Zn^2+^ modulated potent and selective agonists with potential to be developed into oral drugs. Each of 20 selected chemical clusters contained potent compounds displaying DMR GPR39 EC_50_ values < 1 μM with physicochemical properties satisfying the Lipinski rule of five criteria [21] that indicate favorable ADME properties. Also, the three compounds, derived from a limited medicinal chemistry program and selected for *in vivo* profiling, display a high degree of structural diversity. These findings indicate that GPR39 is permissive to binding a broad range of chemotypes for agonistic modulation. This is further supported by recently published Zn^2+^ dependent agonists with yet different chemotypes [22–24]. The druggability of GPR39 is good news and provides an opportunity to further probe the pharmacology of this receptor.

GPR39 couples to Gα_q_, Gα_s_ and Gα_12/13_ pathways. This multiple coupling may be a differentiating advantage for the GPR39 target within the field of diabetes *versus* GPCRs with a more restricted coupling, *e*.*g*. GPR40 (mainly Gα_q/11_) [25] and GPR119 (mainly Gα_s_) [26]. The *in vitro* profiling covered Gα_q_ and Gα_s_ signaling pathways through the IP_1_ and cAMP assays, respectively, and the DMR assay captures the integrated cellular response, including Gα_12/13_ signaling [27]. The IP_1_ and cAMP data shown in Table 1 clearly indicate higher IP_1_/Gα_q_ than cAMP/Gα_s_ potencies in the human GPR39 overexpressing cell lines. The corresponding IP_1_/Gα_q_ potencies in native mouse NIT-1 cells were lower, which may be a consequence of increased number of receptors in the overexpressing system compared to the endogenous GPR39 expressing NIT-1 cells, but also species differences may play a role. It is noted that the IP_1_/Gα_q_ efficacy values in the NIT-1 cells differ dramatically between compounds. The reasons are not known, but may in part be due to potential differences in G-protein composition and GPR39 expression levels between NIT-1 and HEK293 cells. We speculate that the efficacy differences may be physiologically relevant, and that the resolution of efficacy is lost in overexpressing systems. Further work is required to understand efficacy of GPR39 agonists in native cell systems and pathway bias of different agonist series.

GPR39 signaling is activated by Zn^2+^ [6,7], which may also constitute a modulator of an as yet unidentified endogenous GPR39 agonist. Zn^2+^ is regarded as both an agonist and an allosteric modulator of GPR39, and GPR39 is commonly referred to as the zinc sensing receptor. The level of Zn^2+^ in plasma is in the 10 μM range [19,20], but the local and temporal Zn^2+^ concentrations may vary significantly. For example, Herschfinkel *et al*. have suggested a role of GPR39 in neuronal signaling triggered by synaptically released Zn^2+^ [28,29]. Also, with particular relevance for GSIS and the current work, Zn^2+^ is co-released with insulin from islets, and therefore Zn^2+^ modulated agonists may render a particularly potent effect on GPR39 located on the cell surface of insulin secreting cells. This rational is dependent on the assumption that the Zn^2+^ levels by themselves do not saturate GPR39 signaling and its potential effect on GSIS. We therefore regarded the Zn^2+^ modulated agonists identified in the present study as relevant tools to explore the role of GPR39 on GSIS *in vivo*.

The DMR Schild experiments in Fig 4 revealed dramatic Zn^2+^ modulation of all three compounds, with potency increases from about 1000 to 10000-fold going from 0 to 33 μM Zn^2+^. In summary, the Zn^2+^ PAM effect is impressive, generic to several chemotypes and occurs at physiologically relevant Zn^2+^ concentrations.

Interestingly, BSI data suggest that there are two Zn^2+^ binding sites on GPR39. In addition, all three AZ compounds were able to directly bind Zn^2+^. Thus, the multiple GPR39, Zn^2+^ and compound interactions are complex and extensions of the operational model of allosteric agonism and modulation [15] may be required to describe them adequately.

So far, no Zn^2+^ independent GPR39 agonist has been reported. It is tempting to speculate that Zn^2+^-compound complexes interact at one specific site on GPR39. However, more work is required to determine if this site is separate from or involves the N-terminal His^17^ and His^19^ residues previously demonstrated as required for GPR39-Zn^2+^ binding [30].

The screening cascade in the GPR39 project was designed to assess and build a platform of evidence regarding *in vitro—in vivo* correlations with a focus on GSIS. The GSIS *in vitro* experiments in rat INS-1E cells and mouse islets were performed to evaluate if any of the IP_1_/Gα_q_, cAMP/Gα_s_ or DMR potency data would be predictive of GSIS effects. Unfortunately, comparing the INS-1E results in Fig 5 with the data in Table 1, there is no consistent translation between assays. Both AZ7914 and AZ4237 displayed dose dependent GSIS increases in the presence of Zn^2+^ and no effects in the absence of Zn^2+^, in agreement with the screening data shown in Table 1. However, the lack of GSIS effect of AZ1395 was unexpected, considering that AZ1395 was more potent in the IP_1_/Gα_q_ and cAMP/Gα_s_ assays compared to AZ7914 and AZ4237 in the GPR39 overexpressed cells. The slightly lower IP_1_/Gα_q_ potency but greater efficacy of AZ1395 compared to AZ7914 and AZ4237 in native mouse NIT-1 cells may suggest that the IP_1_/Gα_q_ potencies in the NIT-1 cells may be related to GSIS potencies in the rat INS-1E cells.

The mouse islet GSIS experiments showed no significant effect of any of the three compounds, neither in the presence nor in the absence of Zn^2+^. Thus, there was no correlation between rat INS-1E and mouse islet GSIS data. A recent report on a Zn^2+^ dependent GPR39 agonist with potent Ca^2+^/Gα_q_ effects showed no effects on GSIS in human islets and in INS-1E cells [22]. The lack of correlations between assays on recombinant cells and assays on cells endogenously expressing GPR39 is a frustrating dilemma, both regarding drug discovery feasibility and regarding building a pharmacological understanding of the target. In light of the precedence for GPR39 playing a role in GSIS [4], the effects on GSIS in INS-1E cells presented here, and a weak trend between DMR and INS-1E data in the presence of Zn^2+^ (data not shown), glucose tolerance tests *in vivo* were conducted to further explore the role of GPR39 on GSIS.

Three types of *in vivo* experiments were performed. The first *in vivo* IPGTT study was performed on healthy lean mice. None of the three AZ compounds showed any significant insulinotropic effects. In the second study, DIO mice were utilized to assess the effect of GPR39 agonism on GSIS in a disease model. As in lean mice, no compound displayed any insulinotropic effects. Surprisingly, all three AZ compounds showed dose dependent hyperglycemic effects. Zucker fatty rats were tested in the third *in vivo* study, both in order to assess an alternative disease model and to test a second species. The results were similar to those in the DIO mouse study (not shown). The hyperglycemic effects of the GPR39 agonists tested in this study were unexpected in the context of the hypothesis being tested, but it is noted that the role of GPR39 activity in regulating glucose homeostasis has been controversial [12] and the possibility that GPR39 agonism adversely affects insulin sensitivity cannot be discounted. One possibility is that G_s_ signalling triggered by GPR39 causes glucose release from the liver (similar to glucagon and noradrenaline), and high expression of GPR39 in liver has been confirmed [5]. The role of glucagon in the context of potential GPR39 driven hyperglycemia was not explored in this work since GPR39 is absent in alpha-cells [4]. In summary, our *in vivo* studies show that none of the tested GPR39 agonists provide any beneficial increased GSIS effects, neither in normal mice nor in rodent models of β-cell dysfunction.

In order to aid interpretation of the *in vivo* data in the context of GPR39 pharmacology, an analysis of unbound compound exposure *in vivo* in relation to unbound *in vitro* potencies was carried out. The ratios of the compounds average unbound *in vivo* concentrations divided by their unbound *in vitro* EC_50_ values presented in Fig 11 are summarized and interpreted as follows: For the mouse DMR and human cAMP/Gα_s_ data in the GPR39 overexpressing cells in the presence of Zn^2+^, the ratios range from 4.7 to 260 and from 0.81 to 86, respectively, *i*.*e*. ample compound coverage *in vivo* was achieved to conclude that none of these *in vitro* potency parameters translate to a positive GSIS effect *in vivo*. The same interpretation applies to the IP_1_/Gα_q_ data using NIT-1 cells (endogenously expressing GPR39), since the corresponding ratio range was similar (0.30 to 78). The three-point concentration response INS-1E GSIS data allows only for a rough comparison to the *in vivo* exposure data. The *in vivo* exposures of AZ7914 and AZ4237 were 29 and 19 μM, respectively, to be compared with the highest unbound concentrations of 8.4 and 7.2 μM in the INS-1E experiments. Thus, the *in vivo* exposures achieved for these two compounds indicate that the INS-1E effects were not predictive of an *in vivo* effect. All together, the *in vivo* exposure *versus in vitro* potency analysis suggest that none of the DMR, IP_1_/Gα_q_ or cAMP/Gα_s_ *in vitro* potency data generated in the presence of Zn^2+^ are predictive of positive GSIS effects *in vivo*. Future identification of potent and selective GPR39 agonists in the absence of Zn^2+^ would be valuable to further explore the therapeutic potential of GPR39.

## Summary and Conclusion

An HTS based GPR39 agonist lead generation project readily identified 20 Zn^2+^ modulated clusters displaying reasonable physicochemical properties, supporting GPR39 as a druggable target for this mode of action. Following a limited medicinal chemistry program, three structurally diverse compounds were selected as tools to explore the utility of GPR39 agonism on improved GSIS *in vivo*. The lack of positive *in vivo* effects limited the PKPD investigation to an analysis comparing *in vivo* exposure data to *in vitro* potency data. The conclusion from this analysis is that high *in vivo* exposures in relation to the DMR, IP_1_/Gα_q_ and cAMP/Gα_s_ potencies in the presence of physiologically relevant concentrations of Zn^2+^ were not sufficient to yield improved GSIS, neither in normal nor pre-clinical models of β-cell dysfunction. Thus, Zn^2+^ modulated GPR39 agonism does not support this approach to glucose lowering in T2D.

## Supporting Information

S1 Fig IPGTT in Zucker fatty rat. (TIF)

S1 File Chemistry synthesis and experimental details. (PDF)

## Abbreviations

DIO: diet-induced obese
DMF: dimethylformamide
DMR: dynamic mass redistribution
GPCR: G-protein coupled receptor
GTT: glucose tolerance test
HBSS: Hank’s balanced salt solution
HTRF: homogeneous time-resolved fluorescence
IP: intraperitoneal/ly
IPGTT: intraperitoneal glucose tolerance test
KRB: Kreb’s-Ringer bicarbonate
KRH: HEPES-balanced Krebs-Ringer phosphate
PAM: positive allosteric modulator
PKPD: pharmacokinetic-pharmacodynamic
RPMI 1640: Roswell Park Memorial Institute medium 1640
T2D: type 2 diabetes
