# Supplementary material for: Novel Zn2+ Modulated GPR39 Receptor Agonists Do Not Drive Acute Insulin Secretion in Rodents
Source: PLoS One. 2015 Dec 31;10(12):e0145849. doi: 10.1371/journal.pone.0145849 (PMC4697807; doi:10.1371/journal.pone.0145849)
Supplement: S1 File — (PDF) [file pone.0145849.s002.pdf]

SUPPORTING INFORMATION

EXPERIMENTAL PROCEDURES

Chemicals and solvents from commercially available sources was purchased and used without further purification. Purity of all final compounds was 95% or higher at 210 or 230 nm. ^{1}H NMR spectra were recorded on a Bruker biospin GmbH 400, 500 or 600 MHz spectrometer. Chemical shifts are reported in parts-per-million (δ) relative to DMSO-d_6 at 2.50 ppm, $CDCl_3$ at 7.26 ppm or CD_3OD at 3.31 ppm as an internal standard.

Accurate mass was determined using a Waters Acquity UPLC mass spectrometer. Analysis at pH10 on the Waters Acquity system was conducted using a Waters BEH C_{18} 100 mm x 2.1mm, 1.7 µm column. Analysis at pH3 on the Waters Acquity system was conducted using a Waters CSH C_{18} 50 mm x 2.1 mm, 1.7 µm column. The mobile phase used at pH3 contained 10 mM Formic Acid and 1 mM Ammonium formiate, and the mobile phase used at pH10 contained 47 mM Ammonia and 6.5 mM NH_4HCO_3. The gradient was run at 60 °C, the relative absorbance was measured at 210 nm at pH10 and at 230 nm at pH3, and the ionisation mode was ESI+.

Preparation of 6-(3-chloro-2-fluoro-benzoyl)-2-(2-methylthiazol-4-yl)-3,5,7,8-tetrahydropyrido-[4,3-d]pyrimidin-4-one (AZ7914):

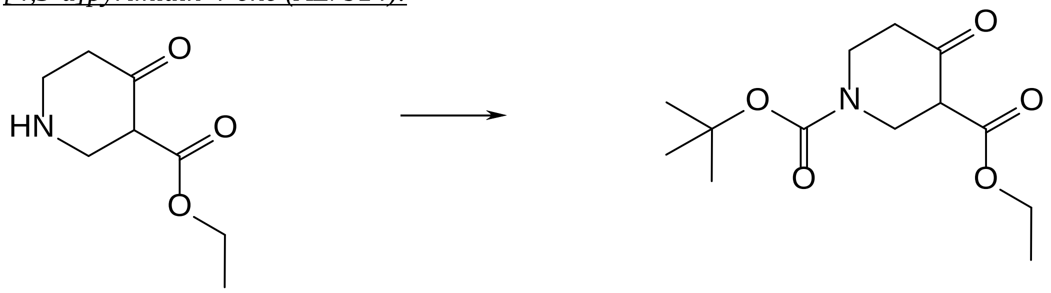

A solution of ethyl 4-oxopiperidine-3-carboxylate x HCl (4.88 g, 23.5 mmol) in dichloromethane (50 mL), triethylamine (6.5 mL) and dimethylaminopyridine (29 mg) was prepared and cooled to 0 °C. Di-tert-butyl bicarbonate (5.13 g) was added to the solution, followed by additional dichloromethane (15 mL). The reaction was stirred at room temperature for 1 h, after which H_2O was added to the solution. The mixture was subsequently filtered through a phase separator and the solvent was evaporated *in vacuo* to give 6.38 g colorless oil. The product was used in the next step without purification.

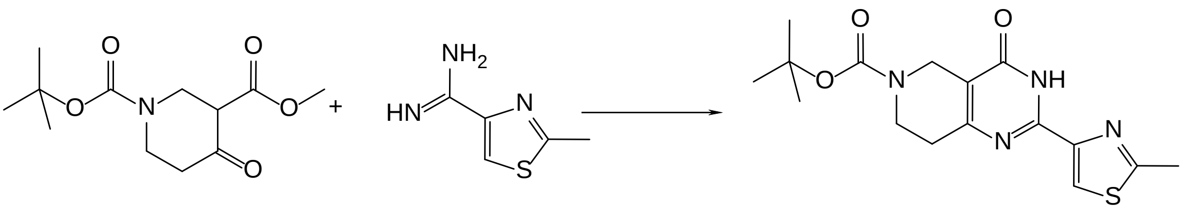

A solution of 2-Methylthiazole-4-carboxamidine hydrochloride (1.27 g, 7.15 mmol) and potassium carbonate (2.470 g, 17.87 mmol) in ethanol (46.4 mL) was prepared. 1-tert-butyl 3-methyl 4-oxopiperidine-1,3-dicarboxylate (2.044 g, 7.15 mmol) was added to the solution, and the reaction mixture was refluxed for 2.5 h. The reaction mixture was cooled to room temperature and ethyl acetate (250 mL) was added. The solution was washed with sodium bicarbonate (saturated, 100 mL) and brine (100 mL). The combined aqueous phases were extracted with ethyl acetate (100 mL). The organic phases were combined, dried using a phase separator and the solvent was evaporated. The crude product was precipitated with DMSO, filtered, and the filtrate was co-evaporated with p-xylene three times to give 1.96 g of a white solid.

^{1}H NMR (500 MHz, CDCl3) δ 1.50 (s, 9H), 2.76 (s, 5H), 3.70 (t, 2H), 4.42 (s, 2H), 8.13 (s, 1H), 10.36 (s, 1H).

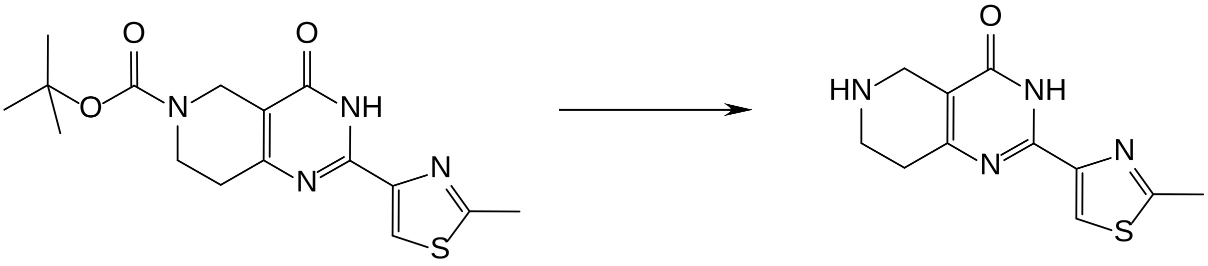

A slurry of tert-butyl 2-(2-methylthiazol-4-yl)-4-oxo-3,4,7,8-tetrahydropyrido[4,3-d]pyrimidine-6(5H)-carboxylate (1.95 g, 5.60 mmol) and 4 M HCl in dioxane (28 mL) in dichloromethane (20 mL) was stirred vigorously over night, after which methanol (20 mL) was added to improve the solubility, and the mixture was reduced *in vacuo* co-evaporating with methanol (3x20 mL). The resulting product was co-evaporated two times with dichloromethane to remove most of the traces of methanol and HCl to yield 2.05 g of product, which was used in the next step without further purification.

^{1}H NMR (400 MHz, MeOD) δ 2.82 (s, 3H), 3.09 (t, 2H), 3.59 (t, 2H), 4.15 (s, 2H), 8.49 (s, 1H).

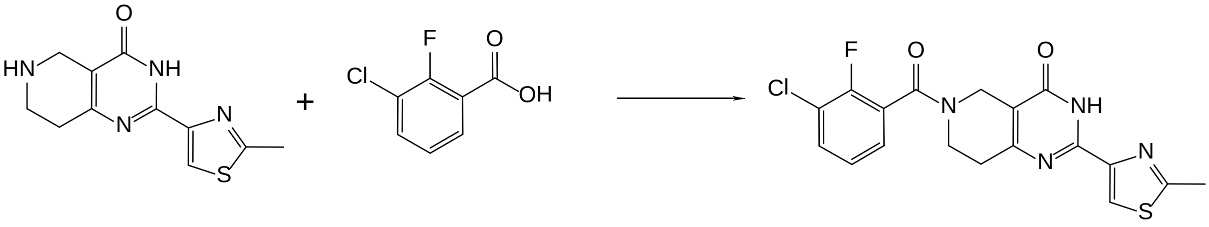

AZ7914

A 250 mL round bottomed flask was charged with 3-chloro-2-fluorobenzoic acid (0.488 g, 2.74 mmol) followed by dichloromethane (10 mL) and N,N'-Diisopropylethylamine (1.002 mL, 5.75 mmol). *o*-Benzotriazol-1-yl-N,N,N',N'-tetramethyluronium tetrafluoroborate (1.055 g, 3.29 mmol) was added at ambient temperature and the reaction was stirred for 5 min. A slurry of 2-(2-methylthiazol-4-yl)-5,6,7,8-tetrahydropyrido[4,3-d]pyrimidin-4(3H)-one (1.000 g, 2.74 mmol) in dimethylformamide (6 mL) and dichloromethane (2 mL), N,N'-Diisopropylethylamine (1.00 mL, 5.75 mmol) was pre-stirred for two min. The mixture was then added to the reaction mixture and the vial from which this was added was rinsed with dichloromethane (8 mL). The reaction mixture turned bright orange and was stirred at ambient temperature for approximately 2 h.

To drive the reaction to completion, an additional amount of 3-Chloro-2-fluorobenzoic acid (0.176 g, 0.68 mmol), *o*-Benzotriazol-1-yl-N,N,N',N'-tetramethyluronium tetrafluoroborate (0.220 g, 0.68 mmol) and N,N'-Diisopropylethylamine (0.487 mL, 2.74 mmol) was added and the reaction was left stirring at room temperature overnight. The reaction was then washed with 8% aqueous sodium bicarbonate (65 mL), followed by H_2O (50 mL), filtered through a phase-separator and the organic layer was concentrated to give 1.509 g of residue as an orange oil. The residue was co-evaporated two times with xylene to give an orange solid (1.141 g). The residue was purified by automated flash chromatography on a 50 g column. A gradient from 0 to 20% ethyl acetate in heptane over one column volume, 20% to 100% of ethyl acetate in heptane over 15 column volumes followed by 100% ethyl acetate over 15 column volumes was used as mobile phase. The product fractions detected in UV at 300 nm were pooled and evaporated. After drying under vacuum for two days, 725 mg of pure product was collected.

^{1}H NMR (400 MHz, DMSO-d_6) δ 2.61 - 2.86 (m, 5H), 3.53 (t, 1H), 3.95 (s, 1H), 4.12 (s, 1H), 4.50 (s, 1H), 7.35 (t, 1H), 7.41 - 7.53 (m, 1H), 7.65 - 7.84 (m, 1H), 8.38 (d, 1H), 12.29 (s, 1H). LCMS accurate mass: calcd $[M+H]^+$ 405.0588, found 405.0602

Preparation of 6-[(4-chlorophenyl)methyl]-7-hydroxy-5-methyl-pyrazolo[1,5-a]pyrimidine-3-carboxylic acid (AZ4237):

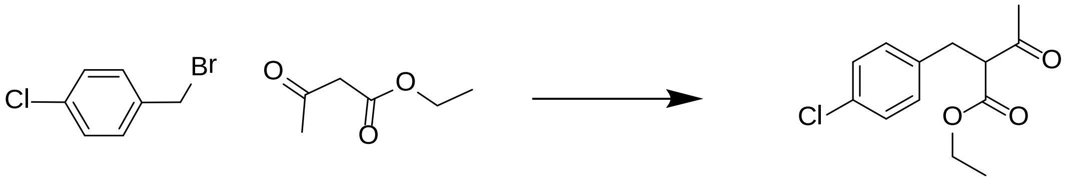

1-(bromomethyl)-4-chlorobenzene (200 mg, 0.97 mmol) dissolved in THF (2 mL) was added to a stirred solution of ethyl 3-oxobutanoate (0.247 mL, 1.95 mmol) and sodium ethanolate (0.153 mL, 1.95 mmol) in THF (2 mL) under nitrogen. The resulting mixture was heated in a microwave reactor at 80 °C for 1 h. H_2O was added and the product was extracted with ethyl acetate. The organic layers were combined and the solvent was removed *in vacuo*. The crude product was used without purification.

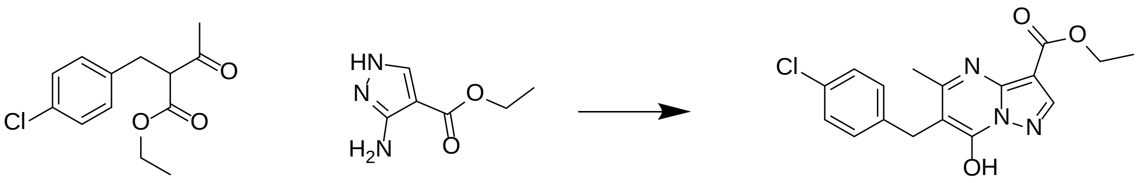

A solution of ethyl 2-(4-chlorobenzyl)-3-oxobutanoate (18.59 g, 72.99 mmol) and ethyl 3-amino-^{1}H-pyrazole-4-carboxylate (11.32 g, 72.99 mmol) in acetic acid (250 mL) was refluxed at 120 °C for 4 h. The solvent was removed *in vacuo*. Ethyl acetate was added and the suspension was stirred at room temperature for 1h. The slurry was filtered, washed and dried to give ethyl 6-(4-chlorobenzyl)-7-hydroxy-5-methylpyrazolo[1,5-a]pyrimidine-3-carboxylate (15.98 g, 63.3 %).

^{1}H NMR (400 MHz, DMSO-d_6) d 1.31 (d, 3H), 2.44 (s, 3H), 3.86 (s, 2H), 4.30 (t, 2H), 7.24 - 7.33 (m, 4H), 8.18 (s, 1H), 11.77 (s, 1H).

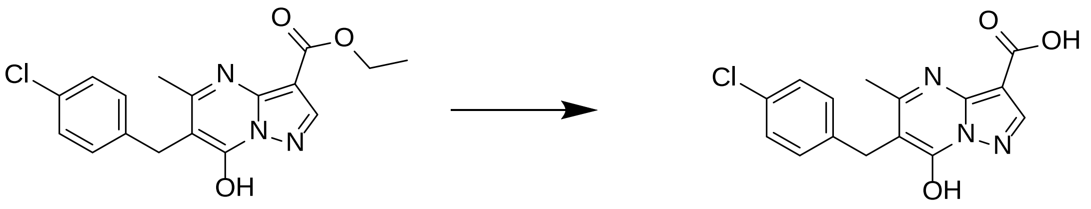

AZ4237

A suspension of ethyl 6-(4-chlorobenzyl)-7-hydroxy-5-methylpyrazolo[1,5-a]pyrimidine-3-carboxylate (4.5 g, 13.01 mmol) and lithium hydroxide (3.12 g, 130.14 mmol) in THF (30 mL) and H_2O (30 mL) was stirred at 70 °C for 5 days. After removal of THF *in vacuo*, the H_2O phase was acidified (HCl). The product was precipitated with ethyl acetate and filtered. The filtrate was washed with H_2O, filtered again and dried *in vacuo* to yield 6-(4-chlorobenzyl)-7-hydroxy-5-methylpyrazolo[1,5-a]pyrimidine-3-carboxylic acid (4.08 g, 99 %).

^{1}H NMR (400 MHz, DMSO-d_6) d 2.43 (s, 3H), 3.86 (s, 2H), 7.24 - 7.32 (m, 4H), 8.15 (s, 1H), 11.69 (s, 1H), 12.76 (s, 1H). LCMS accurate mass: calcd $[M+H]^+$ 318.0645, found 318.0640

Preparation of 3,4-bis-(2-imidazol-1-ylethoxy)benzonitrile (AZ1395):

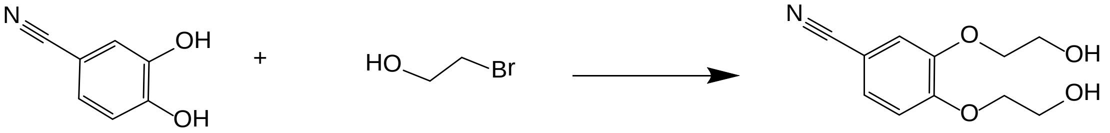

A reaction mixture of 3,4-dihydroxybenzonitrile (1000 mg, 7.40 mmol), 2-bromoethanol (3.15 mL, 44.40 mmol), potassium carbonate (10.230 g, 74.02 mmol) and 18-crown-6 ether (1.174 g, 4.44 mmol) in CH_3CN (30 mL) was degassed and heated for 2 h at 100 °C under microwave irradiation. The reaction mixture was left to cool and was stirred over night. The solvent was then removed under reduced pressure. H_2O and dichloromethane were added and the layers were separated. The aqueous phase was extracted twice with dichloromethane. The combined organic layers were washed with brine, dried using a phase separator and the solvent was removed *in vacuo*. The crude product was used without further purification in the next step.

^{1}H NMR (500 MHz, MeOD) δ 3.88 - 3.93 (m, 4H), 4.09 - 4.16 (m, 4H), 7.09 - 7.12 (m, 1H), 7.3 - 7.35 (m, 2H).

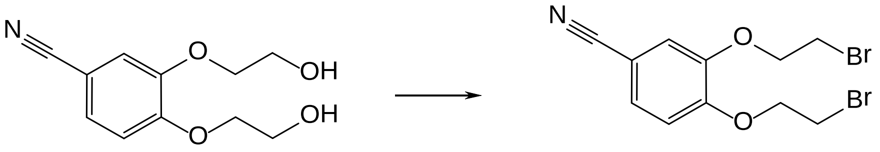

A mixture of 3,4-bis(2-hydroxyethoxy)benzonitrile (2.23 g, 7.49 mmol), triphenylphosphine (7.86 g, 29.97 mmol) and perbromomethane (9.94 g, 29.97 mmol) was dissolved and stirred in dichloromethane (50 mL) at room temperature for 6.5 h. Thereafter, the reaction mixture was concentrated to about one quarter of the volume. The remaining slurry was filtered and the solvent was reduced *in vacuo* to give 10.7 g of an orange foam. The resulting foam was purified using preparative HPLC (Kromasil C8 column, 10 µm 250x20 mm I.D.), using a gradient of 25-65% CH_3CN in $H_2O/CH_3CN/AcOH$ 95/5/0.2 buffer, over 20 min with a flow of 19 mL/min. The compounds were detected by UV at 252 nm.) The relevant fractions were pooled, concentrated, diluted in dichloromethane and dried through a phase separator to afford, after evaporation *in vacuo*, 627 mg of pure product as a white solid.

^{1}H NMR (400 MHz, CDCl3) δ 3.68 (q, 4H), 4.37 (dt, 4H), 6.95 (d, 1H), 7.18 (d, 1H), 7.32 (dd, 1H).

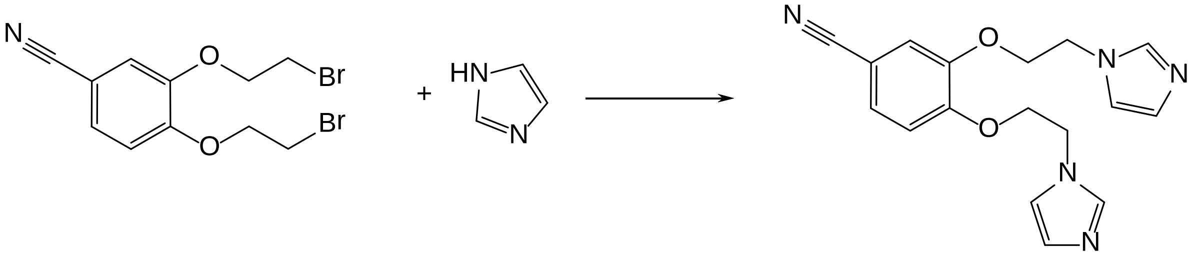

AZ1395

A solution of 3,4-bis(2-bromoethoxy)benzonitrile (626 mg, 1.79 mmol) in dimethylformamide (dimethylformamide) (9.9 mL) was cooled to 0 °C, and ^{1}H-imidazole (488 mg, 7.17 mmol) followed by sodium hydride (313 mg, 7.17 mmol) were added. The reaction mixture was stirred at room

temperature for 1.5 h, after which H_2O was added to quench the remaining sodium hydride. More H_2O was added and the reaction mixture was extracted with dichloromethane (3x). The combined organic phases were washed with brine, dried using a phase separator and the solvent was removed under reduced pressure. Xylene was added to co-evaporate with dimethylformamide to give 700 mg (<90% pure) of a white solid. The resulting solid was dissolved in DMSO to 10 mL and then purified by preparative HPLC (XBridge C18 column, 10 µm 250x19 mm I.D.) using a gradient of 10-50% CH_3CN in $H_2O/CH_3CN/NH_3$ 95/5/0.2 buffer over 20 min with a flow rate of 19 mL/min. The compounds were detected by UV at 254 nm (monitored at 286 nm)). The collected fractions were pooled and evaporated to give 441 mg as an amber oil. The oil was dissolved in CH_3CN and freeze-dried over night to give 440 mg pure product as a white solid.

^{1}H NMR (400 MHz, MeOD) δ 4.21 - 4.37 (m, 4H), 4.4 - 4.54 (m, 4H), 6.89 - 7.13 (m, 3H), 7.13 - 7.39 (m, 4H), 7.72 (d, 2H). LCMS accurate mass: calcd $[M+H]^+$ 324.1.1460, found 324.1465
